# Dataset on the combination effects of 1-methylcyclopropene and salicylic acid on postharvest quality of cut roses ‘Peach Avalanche’ and ‘Sexy Red’

**DOI:** 10.1016/j.dib.2020.106600

**Published:** 2020-11-27

**Authors:** Syariful Mubarok, Masning Maunah, Kusumiyati, Farida, Fathi Rufaidah, Erni Suminar, Ai Yanti Rismayanti

**Affiliations:** a Department of Agronomy, Faculty of Agriculture, Universitas Padjadjaran, Sumedang 45363 Indonesia; b Undergraduate Program of Agrotechnology, Faculty of Agriculture, Universitas Padjadjaran, Sumedang 45363 Indonesia; c Department of Management, ARS University, 40282, Indonesia; d Department of Agrotechnology, Faculty of Agriculture, Universitas Garut, Garut 44151 Indonesia

**Keywords:** 1-MCP, Cut roses, Vase life, Ethylene

## Abstract

Cut roses is one of the important ornamental cut flowers. Many factors affect the loss of postharvest life quality of cut flower, such as temperature, humidity and the presence of ethylene during postharvest storage. In ethylene sensitive roses, ethylene enhances petal discoloration, chlorophyll degradation, petal efflorescence and increase in petal diameter which generating in flower senescence. This data article described the effect of 1-Methylcyclopropene (1-MCP), Salicylic Acid (SA) and its combination on the postharvest life quality of two commercial cut roses varieties, ‘Peach Avalanche’ and ‘Sexy Red’. The flower longevity quality of 1-MCP at 0.5 and 1 µL/L, SA 1.0 and 1.5%, combination of 1-MCP + SA (0.5 + 1.0 µL/L and 1.0 + 1.5%), and untreated flowers were analyzed.

## Specifications Table

**Table:** 

Subject	Agricultural and Biological Sciences
Specific subject area	Agricultural and biological science, and focused on postharvest technology of horticultural crops especially for ornamental plants.
Type of data	Data tables and figure
How data were acquired	This experiment was arranged using a completely randomized design consisting with seven treatments; 1-MCP 0.5 and 1 µL/L, SA 1.0 and 1.5%, combination of 1-MCP + SA (0.5 + 1.0 µL/L and 1.0 + 1.5%), and also without 1-MCP and SA as a control.
Data format	Raw and analysed.
Parameters for data collection	Parameter data on postharvest life quality were collected namely the change of flower color (C, a and b), degree of efflorescence, flower longevity, change of flower diameter, and change of leaf chlorophyll content.
Description of data collection	The experiment was performed in postharvest laboratory at following condition at average temperature of 22 ± 3 °C, 70 ± 5% of humidity and supplemented with 15.000 lm/m^2^ for 12 h. At the beginning and at the end of experiment, the parameters data on postharvest life quality were collected. The obtained data were analyzed using one-way ANOVA, and followed by Duncan's multiple range test (DMRT) at 5%.
Data source location	Institution: Universitas Padjadjaran City/Region: Sumedang Country: Indonesia
Data accessibility	Within the article and on Mendeley Data with the DOI: 10.17632/nhztnsds9v.1, Direct URL to data: https://data.mendeley.com/datasets/nhztnsds9v/1

## Value of the Data

• The data are useful and important for the postharvest handling and treatment of cut roses.
• These data give benefit to scientist, florist and farmers to manage roses cut flower during postharvest handling.
• The data could be used as a basic data for future experiment of cut roses or other ornamental cut flowers.

## 1 Data Description

**Fig. 1:**
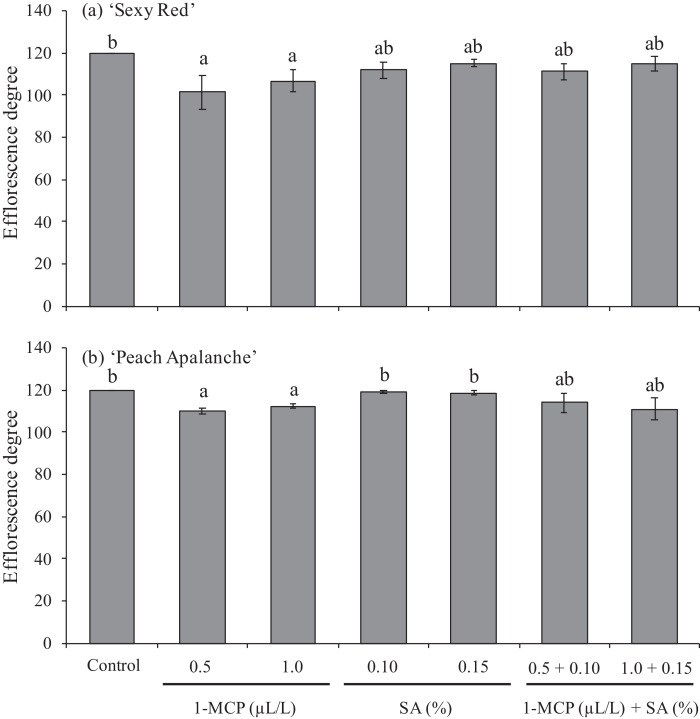
Effect of 1-MCP and SA on the degree of flower efflorescence on (a) ‘Sexy Red’, (b) ‘Peach Avalanche’. The average value ± SE followed by the same letter for each cultivar was not significantly different according to the Duncan Multiple Range Test at the 5% level.

**Fig. 2:**
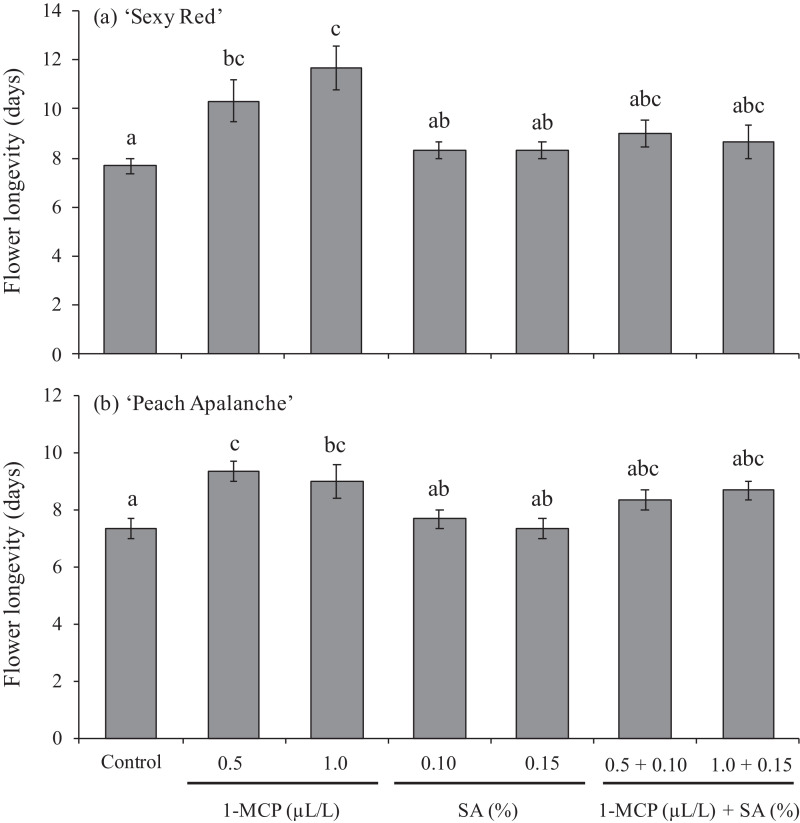
Effect of 1-MCP and SA on flower longevity of (a) ‘Sexy Red’, (b) ‘Peach Avalanche’. The average value ± SE followed by the same letter for each cultivar was not significantly different according to the Duncan Multiple Range Test at the 5% level.

**Fig. 3:**
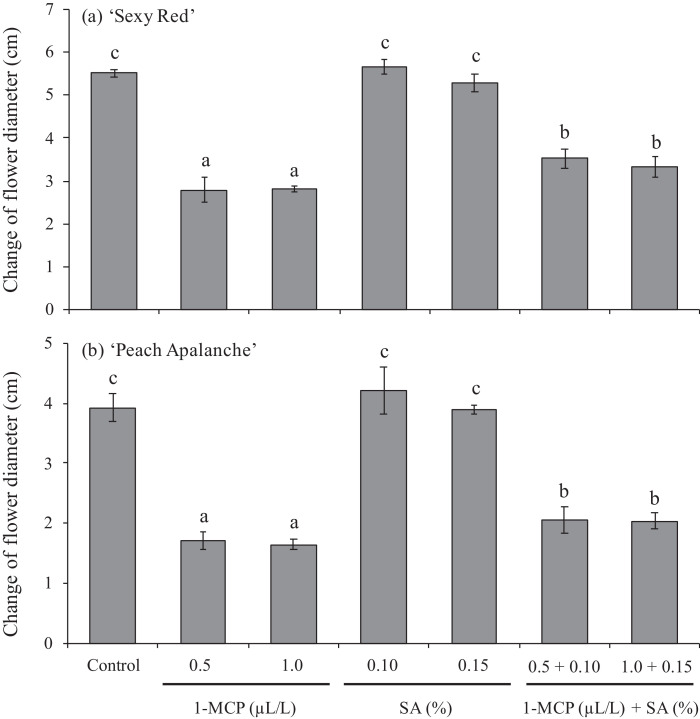
Effect of 1-MCP and SA on the change of flower diameter of (a) ‘Sexy Red’, (b) ‘Peach Avalanche’. The average value ± SE followed by the same letter for each cultivar was not significantly different according to the Duncan Multiple Range Test at the 5% level.

**Fig. 4:**
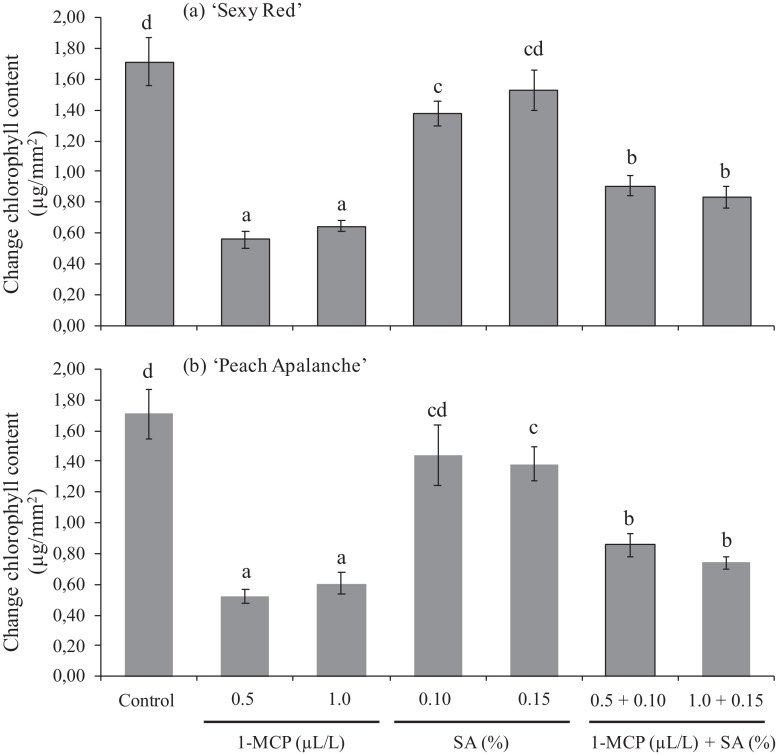
Effect of 1-MCP and SA on the change of chlorophyll content of (a) ‘Sexy Red’, (b) ‘Peach Avalanche’. The average value ± SE followed by the same letter for each cultivar was not significantly different according to the Duncan Multiple Range Test at the 5% level.

The data report the combination effect of 1-Methylcyclopropene and Salicylic Acid on Postharvest Quality of two commercial Cut Roses cultivars, ‘Peach Avalanche’ and ‘Sexy Red’. This information can be used as postharvest treatment of those two roses cultivars to prevent the loss of flower shelf life quality. Data on the color change of petals, *degree of efflorescence,* flower longevity, change of flower diameter, and Change of leaf chlorophyll content are presented in Table 1, HYPERLINK " Figs. 1 –4, respectively. The raw data can be accessed in https://data.mendeley.com/datasets/nhztnsds9v/1.

**Table 1:** The change of flowe color (C, a and b) as an effect of 1-MCP and SA during postharvest storage on ‘Sexy Red’ and ‘Peach Avalanche’.

Treatment	Value		
	∆a*	∆b*	∆C*
**‘Sexy Red’**			
A (control)	−1.69 a	−1.24 a	−1.97 a
B (1-MCP 0.5 µL/L)	4.01 b	3.69 c	4.86 b
C (1-MCP 1.0 µL/L)	4.07 b	3.66 c	4.82 b
D (SA 1.0%)	0.37 ab	0.18 ab	0.42 ab
E (SA 1.5%)	0.25 ab	0.35 ab	0.35 ab
F (1-MCP 0.5 µL/L + SA 1.0%)	1.21 ab	2.16 bc	1.74 ab
G (1-MCP 1.0 µL/L + SA 1.5%)	2.68 ab	1.44 bc	3.04 b
**‘Peach Avalanche’**			
A (control)	−1.04 a	4.46 a	4.05 a
B (1-MCP 0.5 µL/L)	5.17 c	10.69 d	11.83 c
C (1-MCP 1.0 µL/L)	4.58 c	10.57 d	11.49 c
D (SA 1.0%)	−0.61 a	5.52 ab	4.96 a
E (SA 1.5%)	−0.87 a	4.96 a	4.43 a
F (1-MCP 0.5 µL/L + SA 1.0%)	3.45 b	7.39 bc	8.09 b
G (1-MCP 1.0 µL/L + SA 1.5%)	2.10 b	8.72 c	8.96 b

The value followed by the same letter for each cultivar was not significantly different according to the Duncan Multiple Range Test at the 5% level.

### 1.1 The color change of petals

The changes in the color of flower petals from fresh to wilted indicated by the values of ∆a*, ∆b*, and ∆C* after 7 days of storage as an effect of 1-MCP and SA is shown in Table 1. In ‘Sexy Red’, the application of 1-MCP 1.0 and 1.5 µL/L, and also combination of 1-MCP 1.0 µL/L + SA 1.5% significantly increased ∆C* value compared with control (Table 1). The fading of the colors in these three treatments is relatively small and positive, indicating brighter and stronger colors, while the low chroma values in control indicated the duller appearance of ‘Sexy Red’. In ‘Peach Avalanche’, the application of 1-MCP 0.5 and 1.0 µL/L, also combination of 1-MCP 0.5 µL/L + SA 1.0% and 1-MCP 1.0 + SA 1.5% resulted a higher chroma value and significantly different compared with control (Table 1).

Application of 1-MCP 0.5 and 1.0 µL/L resulted the largest difference in ∆a* value and significantly higher compared than control, in ‘Sexy Red’ and Avalanche. but did not significantly different compared to other treatment, except in ‘Peach Avalanche’. In ‘Sexy Red’, application of SA 1.0 and 1.5% and those combination (1-MCP 0.5 µL/L + SA 1.0% and 1-MCP 1.0 µL/L + SA 1.5%) did not show any significant difference compared to the control in the change of ∆a*, but in Peach Avalanche only SA 1.0 and 1.5% that showed a similar ∆a* value compared with control (Table 1). Application of 1-MCP 0.5 and 1.0 µL/L resulted the largest difference in ∆b* value and significantly higher compared than control, in ‘Sexy Red’ and Avalanche but did not significantly different compared to 1-MCP 0.5 µL/L + SA 1.0% and 1-MCP 1.0 µL/L + SA 1.5% treated flower. However, the application of SA 1.0 and 1.5% did not show any significant effect in maintain ∆b* value compared to control in both cultivars (Table 1).

### 1.2 The degree of efflorescence

The results of data analysis measuring the efflorescence degree of ‘Sexy Red’ and ‘Peach Avalanche’ varieties at during storage can be seen in Fig. 1. In the control treatment, the ‘Sexy Red’ and ‘Peach Avalanche’ varieties resulted a relatively short efflorescence time, reaching a maximum value of degree of efflorescence of 120° at the end posthrvest storage (Fig. 1). The application of 1-MCP 0.5 and 1.0 µL/L significantly inhibit an efflorescence degree of flower up to 18.42° and 13.17° lower than control, respectively, in ‘Sexy Red’ and up to 9° lower than control in ‘Peach Avalanche’. However, the application of SA 1.0 and 1.5% and also those combinations did not show any significant effect in ‘Sexy Red’ and ‘Peach Avalanche’ in inhibiting flower efflorescence (Fig. 1).

### 1.3 Flower longevity

Based on statistic data analysis showed that application of 1-MCP 0.5 and 1.0 µL/L was effective in increasing flower longevity of two investigated cut roses cultivars, ‘Sexy Red’ and ‘Peach Avalanche’. Those 1-MCP concentrations, 0.5 and 1.0 µL/L, significantly extended flower longevity up to 3.33 and 4.67 days, respectively, longer that control for ‘Sexy Red’, and up to 2.33 and 1.67 days, respectively, longer that control for ‘Peach Avalanche’. However, combination treatment of 1-MCP and SA, and also SA alone, did not affect in increasing flower longevity on two investigated cut roses (Fig. 2).

### 1.4 Change of flower diameter

The results of statistical analysis through the Duncan test at 5% level showed the effect of 1-MCP and SA solutions on the change of flower diameter of the ‘Sexy Red’ and ‘Peach Avalanche’ cultivars. Average data is presented in Fig. 3. The changes in increasing flower diameter of the two investigated roses cultivars, ‘Sexy Red’ and ‘Peach Avalanche’”, can be effectively suppressed by the application of 1-MCP and its combination with SA. According to the statistical data analysis showed that application 1-MCP 0.5 and 1 µL/L and also 1-MCP 0.5 µL/L + SA 1% and 1-MCP 1 µL/L + SA 1.5%, resulted the significant lower value of increasing of flower diameter compare than control, but the application SA only at 1 and 1.5% did not affect in inhibit the increasing of the change flower diameter (Fig. 3).

### 1.5 Change of leaf chlorophyll content

According to statistical data analysis using Duncan Multiple Range Test at 5% showed that the change of chlorophyll content presented extremely diverse values between the two cultivars. 1-MCP 0.5 and 1.0 µL/L threated flowers presented the low change value in chlorophyll with a value of 0.56 and 0.71 μg/mm^2^, respectively in ‘Sexy Red’, and 0.52 and 0.61 μg/mm^2^, respectively, in ‘Peach Avalanche’ (Fig. 4). The application of 1.0 µL/L and 1.5% of Salicylic acid in the ‘Sexy Red’ and ‘Peach Avalanche’ cultivars did not effective in maintaining leaves chlorophyll content and did show any significant differences compared to control. However, on both cultivars, the combination effect of 1-MCP and SA can maintain leaves chlorophyll content and shows a significant chlorophyll content compared to control (Fig. 4)

## 2 Experimental Design, Materials and Methods

### 2.1 Flower preparation

Two rose cultivars were obtained from the Grace Farm in Parongpong, Bandung, Indonesia. The flowers were harvested by cutting the flower stems ± 65 cm with the flower criteria were have an efflorescence of 10% i.e. the outer petal (1–2 strands of petal) opens slightly and the inner of petal is still tightly closed. The harvested flowers were then transported in the water to the Postharvest Laboratory of the Faculty of Agriculture, Universitas Padjadjaran. Before being given 1-MCP and SA, the flower stalk was recut under water to get a 55 cm long flower stalk with five leaves. The experiments were conducted in a completely randomized block design that consists of seven treatments combination: tap water (control); 1-MCP 0.5 and 1.0 µL/L without SA; SA 1.0 and 1.5% without 1-MCP; 1-MCP 0.5 µL/L with SA 1.0%; and 1-MCP 1.0 µL/L with SA 1.5%. Those treatment were replicated three times and consisted of five cut flowers per treatment. 1-MCP was applied using fumigation method in a sealed glass chamber. The flowers were placed in three different glass chamber namely untreated 1-MCP (control), 1-MCP 0.5 and 1.0 µL/L. After 18 hours incubation, the chambers were ventilated and the 1-MCP treated flower transferred into 1 L glass bottle containing SA holding solution (1.0% and 1.5%) and tap water as control and placed on the laboratory bench at following condition at average temperature of 22 ± 3 °C, 70 ± 5% of humidity and supplemented with 15.000 lm/m^2^ for 12 h.

### 2.2 Petal color analysis

Three petals were taken from each flower for color measurement. The flower color was measured at the beginning of storage (day 0) and repeated at the flower quality was lost in the vase-life by using a Minolta Chroma Meter (Model CR-300, Minolta, Osaka, Japan). Color measurements were taken from the left, right and center of petal. Three parameters of color were giving from this device, they were brightness (L), red to green scale (a) and yellow to blue scale (b). From this result, the saturation index of chroma (C) was calculated using the equation: of $C=\sqrt{\left(a^{2}+b^{2}\right)}$

### 2.3 The degree of efflorescence

**Fig. 5:**
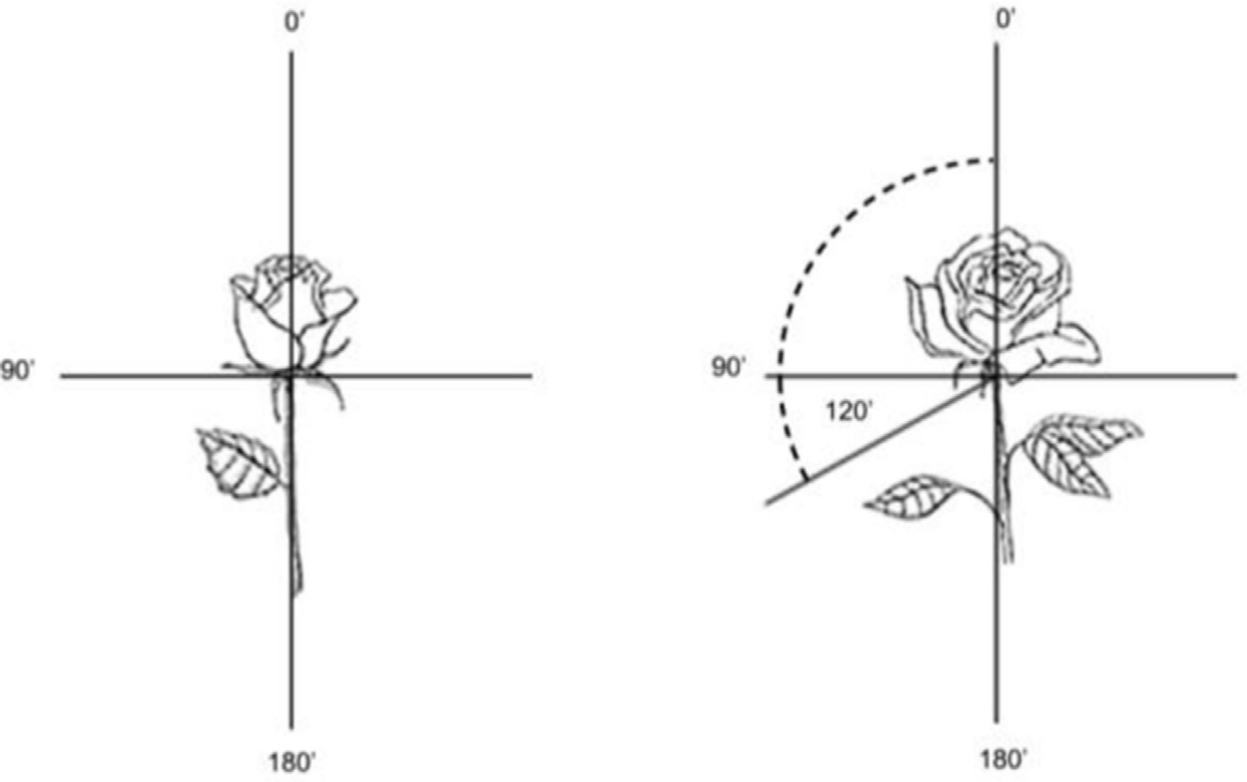
Simulation for to determine degree of flower efflorescence.

One of the requirements for quality of cut roses is an upright petal. During the senescence process, the lowest petal will continue to droop and signify the reduced quality of the cut flower. The degree of efflorescence was carried out using an arc by measuring the angle of the petal curl from 0° to the horizontal line of the stem with a maximum degree of petal curl of 120° (Fig. 5).

### 2.4 Change of flower diameter

Flower diameter were measuring by using calipers. The measurement was carried out at the beginning of storage and at the end of storage time. Flower diameter was measure at two sides of widest and shortest diameters of the flower crown. The changes in flower diameter were calculated by subtracting the data on day-n after treatment with data on day-0 as following equation ∆*D* = *Dn* - *D*_0_, where ∆*D* is the change in flower diameter, *Dn* is the flower diameter data on the day-n (diameter after n days of storage time), and *D*_0_ are flower diameter data on day 0 (initial diameter at the beginning of storage).

### 2.5 Flower longevity

Flower longevity was measured by counting according to method described by Afiifah et al. [1] and Mubarok et al. [2] by counting the number of days starting from the time of harvest until the quality of flowers decreases, which was indicated by the degree of efflorescence has reached ≥ 120° (Fig. 5).

### 2.6 Change of leaf chlorophyll content

Chlorophyll analysis was performed according to Lichtenthaler [3]. At the beginning, the fresh masses of leaves without petiole were weighed. Three 8 mm diameter discs were excised from the left, center and right of each leaf blade using a cork borer and then inserted into a 1.5 ml reaction tube. The excised leaves were extracted with 1 mL of 80% ethanol at 75 °C for 10 min. Absorption was measured using an Orion AquaMate 8000 UV–vis Spectrophotometers (Thermo Scientific, USA) at 647, 664 and 700 nm. The equation for the determination of the concentration of chlorophyll_a+_*_b_* content was:

$$\text{Chlorophyl}l_{a+b}\left(\text{Ch}\right)=5,24\times \left(A_{664}-A_{700}\right)+22\times \left(A_{647}-A_{700}\right).$$

The data was expressed as a change of chlorophyll content(μg/mm^2^)

### 2.7 Statistical data analysis

The data are represented as the mean values ± SE of three replicates. For the statistical data analysis, data were subjected to one factor analysis of variance (ANOVA) followed by Duncan Multiple Range Test (DMRT) at *p* < 0.05 for comparisons among the investigated data.

## Declaration of Competing Interest

The authors declare that they have no known competing financial interests or personal relationships which have, or could be perceived to have, influenced the work reported in this article.
